# Advance directives among cognitively impaired persons who had an amyloid PET scan and their care partners: a mixed-methods study

**DOI:** 10.1186/s12904-022-01082-4

**Published:** 2022-11-06

**Authors:** Emmanuelle Bélanger, Elyse Couch, Michaela S. Carroll, Nicole DePasquale, Emily A. Gadbois, Megan Shepherd-Banigan, Eric Jutkowitz, Courtney H. Van Houtven, Brenda L. Plassman, Terrie T. Wetle

**Affiliations:** 1 Center for Gerontology and Healthcare Research, Brown University School of Public Health, 121 South Main Street, 6th Fl., Providence, RI 02903 USA; 2 Department of Health Services, Policy & Practice, Brown University School of Public Health, Providence, RI USA; 3 Division of General Internal Medicine, Duke University School of Medicine, Durham, NC USA; 4 Department of Population Health Sciences, Duke University School of Medicine, Durham, NC USA; 5 Duke-Margolis Center for Health Policy, Durham, NC USA; 6 Center of Innovation to Accelerate Discovery and Practice Transformation, Durham VA Health Care System, Durham, NC USA; 7 Department of Psychiatry and Behavioral Sciences, Duke University School of Medicine, Durham, NC USA; 8 Department of Neurology, Duke University School of Medicine, Durham, NC USA

**Keywords:** Advance directives, Alzheimer’s disease, Plaques, amyloid, Cognitive impairment

## Abstract

**Background:**

Little research exists on the role of β-amyloid PET scans as part of Alzheimer’s diagnostic tests and documentation of end-of-life preferences for persons with cognitive impairment. The study objectives were to examine the association of amyloid PET scan results (elevated vs. not elevated amyloid levels) and diagnostic category (mild cognitive impairment vs. dementia) with the likelihood of having an advance directive (reported a median of 4.5 months post-scan); to explore perceptions of PET scan results and their influence on planning for the future among persons with cognitive impairment and their care partners.

**Methods:**

Sequential, explanatory mixed-methods design using data from dyads in the CARE-IDEAS study: advance directives as a factor of diagnostic category and scan result using multivariable logistic regression models; thematic analysis of semi-structured interviews with persons with cognitive impairment and care partners to explore how scan results influenced documentation of future healthcare preferences. Participants included 1784 persons with cognitive impairment and care partners from the CARE-IDEAS study, and a subsample of 100 semi-structured telephone interviews.

**Results:**

81.6% of dyads reported an advance directive. Non-Hispanic, White participants had higher rates of advance directives. There was no significant association between having an advance directive and scan results. Qualitative analysis provided insight into perceived urgency to have advance directives, evolving healthcare preferences, and the context of completing advance directives.

**Conclusions:**

Although amyloid PET scans prompted persons with cognitive impairment and care partners to consider progressive cognitive impairment as part of evolving healthcare preferences, we found substantial variability in the perceived urgency of documentation.

**Supplementary Information:**

The online version contains supplementary material available at 10.1186/s12904-022-01082-4.

## Introduction

In 2015, 46.2 million people worldwide lived with dementia, a number projected to grow to nearly 131.5 million by 2050 [1]. The most common cause of dementia is Alzheimer’s disease, a progressive terminal illness affecting neurons involved in cognitive processes that eventually inhibits basic functioning [2]. Diagnosing Alzheimer’s disease and related dementias is a multi-step process and can involve a variety of tests, including amyloid positron emission tomography (PET) scans. Beyond establishing eligibility for novel pharmacological interventions in a research setting [3–5], increased diagnostic accuracy conferred by amyloid PET can potentially improve healthcare planning for patients and families and has been found to change the medical management of patients [6]. Amyloid PET scans assess the level of Aβ plaques in the brain, with a normal level excluding a diagnosis of Alzheimer’s disease and indicating dementia is caused by other disease processes [7, 8]. Planning for the future is particularly important for persons with Alzheimer’s disease and related dementias, given the uncertain prognosis and potentially extended decline with diminished capacity necessitating reliance on surrogate decision-makers.

Individuals can make their preferences for future care known through advance care planning, a process that may include different elements for different individuals, and may change as illness progresses [9]. Advance care planning can take the form of discussing one’s wishes with family members, caregivers, and healthcare providers, and/or the completion of written advance directives in medical records [9], in anticipation of a time at which the patient may no longer have capacity. As a component of advance care planning, durable power of attorney for healthcare allows individuals with progressive dementia to prepare for future healthcare decisions. A durable power of attorney for healthcare is a specific designation made by a patient to put the decision-making power in the hands of another individual when the patient is unable to do so themselves, and is particularly important in cases where the prognosis includes future incapacity, as is likely for persons with Alzheimer’s disease and related dementias [10]. A recent systematic review [11] concluded that advance care planning supports future autonomy and is associated with positive end-of-life outcomes, and advance directives are also a clinically useful tool for practice and evaluation [12]. Advance directives can offer clarity for healthcare providers and surrogate decision-makers, and improve concordance in end-of-life care [13].

Despite the benefits of advance directives, there are many barriers to advance care planning for persons with dementia, including difficulty predicting their progression [13, 14]. They may also struggle with advance care planning because they do not want to consider a future in which they are unable to make decisions for themselves, or feel powerless to change what feels like a bleak prognosis [15, 16]. Knowing when to begin and revisit conversations about the future and preferences for care is another barrier to advance care planning [14, 16–19]. In previous qualitative work, healthcare professionals described discomfort initiating conversations about advance care planning and being unsure of the “best” time to do so, particularly if patients themselves are unaware of the urgency [19, 20]. Persons with dementia and their carers also reported difficulty identifying the “right” time for advance care planning, describing the point of diagnosis as “too soon” but struggling to pinpoint an appropriate time [14]. Alzheimer’s disease is both progressive and terminal, and the challenge lies in finding a time when patients and families are able to consider an uncertain future but before the patient has lost capacity to make these decisions [16].

Previous research has not explored how amyloid PET scan results may influence the completion of advance directives and plans for the future, and it is possible the disclosure of amyloid scan results may present the “right time” to engage in advance care planning and prompt discussion among patients, care partners, and healthcare professionals. Advance care planning is not a single discussion, but beginning the process can be a difficult step. With increasingly advanced diagnostic technology it is vital to understand patient and care partner perspectives on the role amyloid scan results play in completing an advance directive. The objective of this mixed-methods study was to examine the relationship between amyloid PET scan results and diagnostic category at enrollment (mild cognitive impairment vs. dementia) with the likelihood of having an advance directive among a sample of Medicare beneficiaries with memory issues of unknown etiology. We aimed to better understand how scan results are interpreted by persons with cognitive impairment and their care partners in the context of advance care planning. The research questions guiding our quantitative investigation were: How likely are persons with cognitive impairment who had an amyloid PET scan to have an advance directive, and to what extent does this vary by diagnostic category and scan result? We hypothesized that persons with dementia and those with elevated amyloid would be more likely to report an advance directive. For the qualitative portion of the study, we investigated the role amyloid scan results play in the documentation of end-of-life care preferences among persons with cognitive impairment and their care partners.

## Methods

### Mixed-methods research design

**Fig. 1:**
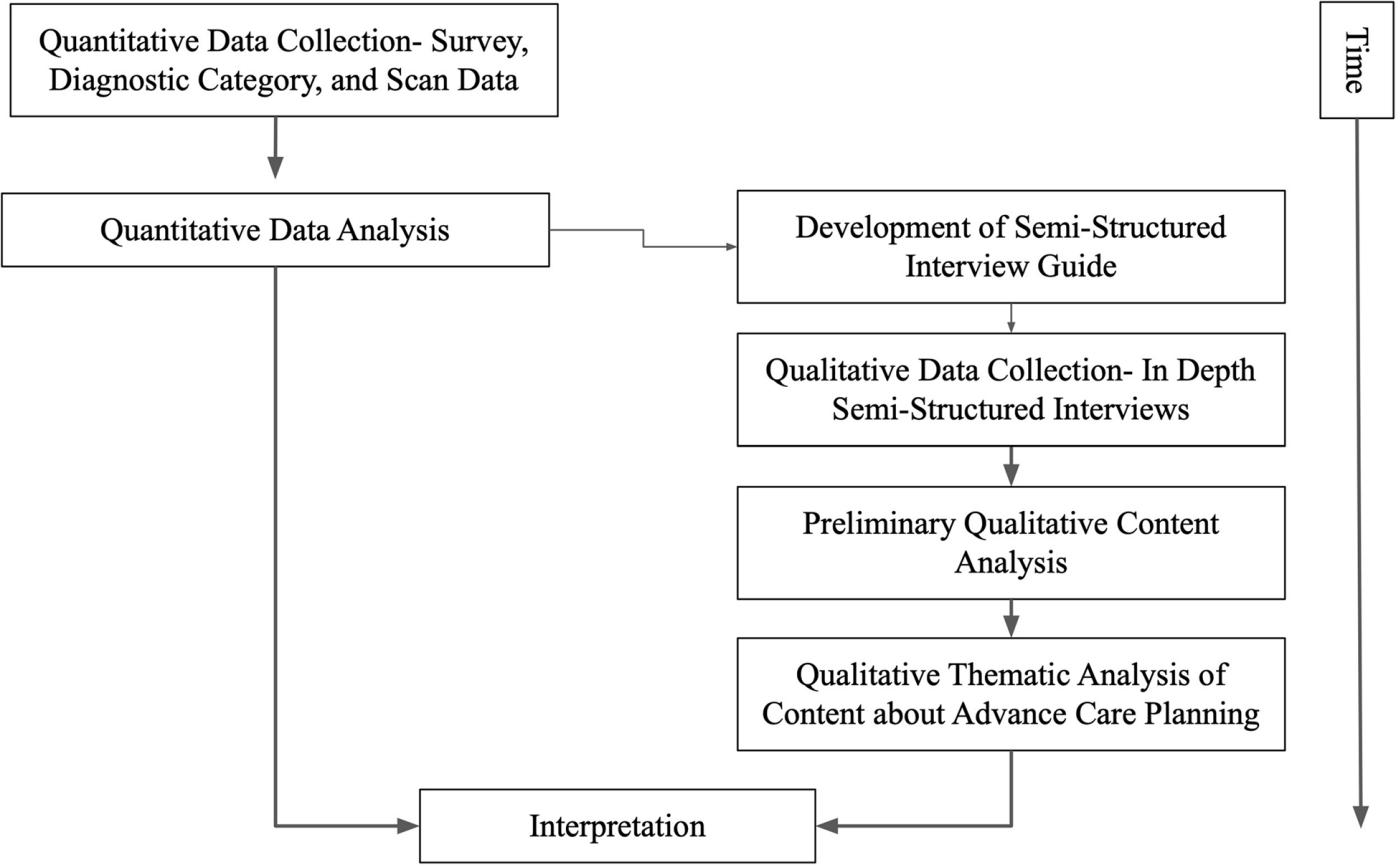
Mixed-methods research design

This study consists of a sequential, explanatory mixed-methods design [21] to better understand the role that amyloid PET scan results play in planning for the future and completing advance directives according to persons with cognitive impairment and the family members and/or friends who care for them (referred to as care partners). Quantitative data collection and analysis preceded and informed the subsequent qualitative stage, and generated additional explanatory data and insight (see Fig. 1). Mixing of methods occurred during qualitative data collection, analysis, and interpretation. Semi-structured interview questions deepened our understanding of the influence of PET scan results on participants’ plans for the future and completion of advance directives. Quantitative findings informed stratification of qualitative data for analysis, shedding light on participants’ perspectives about advance directives given their memory issues and amyloid PET scan results.

### Quantitative methods and measures

The quantitative analyses rely on baseline survey data from the “Caregivers’ Reactions and Experience, a supplemental study of the Imaging Dementia Evidence for Amyloid Scanning Study (CARE-IDEAS),” which recruited a sub-sample of 2228 IDEAS participants and 1872 of their care partners. The IDEAS study examined change in the clinical management of 16,008 Medicare beneficiaries with cognitive impairment of uncertain etiology who underwent an amyloid PET scan between 2016 and 2017 as part of diagnostic procedures for Alzheimer’s disease [6]. As described by the IDEAS protocol, amyloid PET results were discussed in accordance with routine clinical practice across the 592 participating dementia practices and the discussion was not standardized by the trial. The procedures and survey instruments employed in the CARE-IDEAS study have been described in previous publications [22–25]. Briefly, the IDEAS study team provided contact information of IDEAS patients who agreed to be contacted for supplemental studies (*N* = 12,474) to TrialMatch®, the Alzheimer’s Association’s clinical studies matching service. TrialMatch® agents then recruited participants into supplemental studies, including CARE-IDEAS. Among the 3717 IDEAS study patients contacted for the CARE-IDEAS study, 2228 persons with cognitive impairment and 1872 of their care partners completed the baseline survey. The CARE-IDEAS study was approved by the Brown University Institutional Review Board (Protocol#1606001534) on June 24th, 2016. All methods were carried out in accordance with relevant guidelines and regulations.

Structured survey items were administered by telephone in 2017-2018. The median time between receiving the amyloid PET scan and taking the baseline CARE survey was 4.5 months with an interquartile range of 2.3-9.3 months. The main outcome of interest for this manuscript was the self-reported presence of an advance directive, assessed by asking persons with cognitive impairment: “Do you have an advance directive? This document describes your preferences for resuscitation and other life-saving procedures;” and asking care partners: “Does <PATIENT> have an advance directive? This document describes <HIS/HER> preferences for resuscitation and other life-saving procedures.” Our final analytical sample was restricted to dyads in which both members completed the telephone interview and answered whether or not the person with cognitive impairment had an advance directive, resulting in 95.3% of the eligible population (*N* = 1784). Given that persons with cognitive impairment may not remember having an advance directive and/or care partners may not know about existing ones, we considered the person with cognitive impairment as having an advance directive if either they or their care partner reported one, to lower the rate of missing values. We conducted sensitivity analyses to determine whether using only the care partner report would change our results.

The main independent variables of interest were elevated or not elevated amyloid levels based on the PET scan, and whether the person with cognitive impairment was identified as having dementia or mild cognitive impairment at the time of the scan. Covariates known to have a significant association with the completion of advance directives were included in fully adjusted models, including age (below 65, 65-74, 75-84, 85+), gender (man or woman), education (less than high school diploma, some college, college degree, postgraduate degree), and self-reported race/ethnicity (Non-Hispanic White, Hispanic White, non-Hispanic Black, or other) [10, 26, 27].

We first examined self-reported advance directive completion rates using descriptive statistics. We then estimated univariate regression models for the two main variables of interest, and finally added covariates to fully-adjusted multivariable logistic regression models assessing the relationship between diagnostic category, scan results, and likelihood of having an advance directive. Regression models were estimated separately based on respondent and care partner variables given high correlation between these characteristics across our dyads. The majority of the dyads in the CARE-IDEAS study were spouses.

### Qualitative data collection and analysis

In-depth qualitative interviews were conducted via telephone between May 2020 and January 2021 by two funded female graduate research assistants and one funded female, PsyD-trained research coordinator under the supervision of team members (EB, EG, TFW). The interview guide (see Additional file 1: Appendix) comprised various open-ended questions with probes to obtain participants’ perceptions of amyloid PET scan results and how they may have influenced their plans for the future and was pilot tested with 10 participants prior to completion of the full sample. Participants were eligible if they had completed CARE-IDEAS surveys, agreed to be contacted again for future studies, and had a score of at least 21 on the Modified Telephone Interview for Cognitive Status (M-TICS) conducted as part of the CARE-IDEAS study within the previous year. A score ≥ 21 indicated sufficient cognitive ability to meaningfully participate in a semi-structured interview [28]. Participants were mailed a consent form and then contacted by research assistants with whom they had no prior relationship for a single in-depth telephone interview lasting approximately 30 to 60 minutes. After reviewing the information provided in the consent form, covering goals of the research, risks and benefits, and other relevant information together, participants were prompted to explain the purpose of the study in their own words as an additional screening of cognitive ability. Verbal informed consent was obtained from all participants. Given that the CARE-IDEAS study cohort was relatively homogenous in terms of race and ethnicity, we oversampled from participants who had not self-identified as non-Hispanic White in the baseline survey to ensure greater diversity of perspectives. We stratified recruitment by diagnostic categories and amyloid PET scan results to obtain a similar population of participants to that of the overall CARE-IDEAS study. This qualitative follow-up study was approved by the Institutional Review Board at Brown University (Protocol #1606001534) on February 14th, 2020.

Interviews were audio recorded with permission, transcribed verbatim, and imported into NVivo [29] and were not returned to participants for comment. Interviewers made field notes for the interviews to add clarity. We used exploratory content analysis to identify relevant content in a large corpus of data, based on an iterative coding book developed after coding 10 interviews with a team of five researchers. All interviews were then coded by the first author according to this coding book, and the remaining team members verified the coding for accuracy. No new relevant findings emerged through the remaining coding process. Any discrepancies in coding across analysts were discussed until consensus was reached. Participants were not invited to offer feedback on the findings. The first and second authors imported stratifying survey data into NVivo and used query functions to perform thematic analysis [30] about the meaning of the PET scans for the future and the process of care preference documentation along relevant dimensions. Variables chosen for stratification were derived from the results of the quantitative analyses, namely amyloid PET scan results, age, education, and race/ethnicity. Thematic analysis involved reading through query results independently and discussing observations before abstracting larger patterns from the data as relevant to the research question. The research team kept an audit trail documenting the analytical process to maintain a transparent record of study decisions.

## Results

### Quantitative sample description

Our quantitative sample included 1872 dyads who completed the baseline structured surveys for the CARE-IDEAS study. Of this cohort, 88 were excluded from analyses due to missing values for variables of interest. As seen in Table 1, the majority of persons with cognitive impairment (60.8%) were men, and 93.9% self-identified as non-Hispanic White. Over half (52.6%) were between 65 and 74 years of age. For comparison, the parent IDEAS study reported 11,409 participants with a complete record, 60.5% of which had MCI, and 39.5% had dementia, with 61.1% of the overall sample having elevated amyloid plaques. As can be expected with longitudinal follow-up of participants willing to be contacted again, our CARE-IDEAS sample was slightly less impaired (73.6% MCI and 26.4% dementia), but a larger percentage had elevated amyloid (68.5%). Slightly less than half of care partners were between 65 and 74 years of age (47.2%). The majority (67.8%) of care partners were women, and 93.8% self-identified as non-Hispanic White. The vast majority (88.4%) of dyads were married or partnered and living together. Both persons with cognitive impairment and care partners were overall highly educated, with 58.7% of respondents and 57.5% of care partners having a Bachelor’s or graduate degree.

**Table 1:** Descriptive characteristics of study dyads *N* = 1784

	Persons with Cognitive Impairment N (%)	Care Partners of Persons with Cognitive Impairment N (%)
**Characteristic**		
**Age- n (%)**		
Below 65	N/A	341 (19.11)
65-74	938 (52.58)	842 (47.20)
75-84	762 (42.71)	549 (30.77)
85+	84 (4.71)	52 (2.91)
**Gender - n (%)**		
Men	1079 (60.48)	574 (32.17)
Women	705 (39.52)	1210 (67.83)
**Race / Ethnicity- n (%)**		
Non-Hispanic, Caucasian	1675 (93.89)	1674 (93.83)
Non-Hispanic, African American	30 (1.68)	29 (1.63)
Hispanic, Caucasian	42 (2.35)	38 (2.13)
Other	37 (2.07)	43 (2.41)
**Education- n (%)**		
Secondary or less	280 (15.70)	257 (14.41)
Vocational / some college	456 (25.56)	499 (27.97)
Bachelor’s degree	422 (23.65)	484 (27.13)
Graduate degree	626 (35.09)	544 (30.49)
**Diagnostic Category- n (%)**		
Mild Cognitive Impairment	1313 (73.60)	N/A
Dementia	471 (26.40)	N/A
**β-Amyloid Scan Results- n (%)**		
Not elevated	562 (31.50)	N/A
Elevated	1222 (68.50)	N/A

### Quantitative findings

The overall rate of self-reported advance directives in our sample was relatively high; in 81.6% of dyads, at least one person reported that the person with cognitive impairment had an advance directive at the time of the CARE-IDEAS telephone survey interview. Concordance was present in 77.4% of dyads, with 61.5% both reporting the presence of an advance directive, and 15.9% agreeing about its absence. When members of the dyad disagreed, the person with cognitive impairment reported advance directives and the care partner did not in 5.3% of the analytical sample, while 9.3% had the reverse scenario. In the rest of the dyads (8.0%), one member reported not knowing about advance directives. Contrary to our hypothesis, there was no significant association between having an advance directive and either diagnostic category at enrollment or amyloid PET scan results. In fully-adjusted, multivariable models (see Table 2), the following socio-demographic characteristics were associated with a higher likelihood of having an advance directive: being 75-84 years old, male respondent gender or female care partner gender, non-Hispanic White race/ethnicity, and higher education level. Race/ethnicity was an important covariate; persons with cognitive impairment identifying as a race or ethnicity other than non-Hispanic White were less than half as likely to have an advance directive. Sensitivity analyses using only care partner report yielded similar conclusions, with 76.9% of dyads in the sample of 1706 where care partner report was available having an advance directive, and the same significant associations with socio demographic characteristics.

**Table 2:** Odds ratios of reporting an advance directive as a factor of participant characteristics, *N* = 1784 (95% confidence intervals)

	Persons with Cognitive Impairment		Care Partners	
	Univariate	Multivariable	Univariate	Multivariable
**Age**				
Below 65	N/A	N/A	Ref	Ref
65-74	Ref	Ref	1.30 (.95, 1.76)	1.27 (.93, 1.75)
75-84	1.61 (1.25, 2.08)	1.53 (1.18, 1.99)	1.72 (1.22, 2.42)	1.83 (1.27, 2.63)
85+	1.67 (.89, 3.13)	1.57 (.82, 2.98)	1.66 (.75, 3.67)	1.90 (.84, 4.29)
**Gender**				
Men	Ref	Ref	Ref	Ref
Women	.66 (.52, .84)	.75 (.58, .97)	1.40 (1.09, 1.80)	1.63 (1.25, 2.12)
**Race / Ethnicity**				
Non-Hispanic, White	Ref	Ref	Ref	Ref
Non-Hispanic, African American	.21 (.10, .43)	.22 (.10, .46)	.34 (.16, .72)	.35 (.16, .76)
Hispanic, White	.41 (.22, .80)	.47 (.24, .91)	.35 (.18, .69)	.36 (.18, .71)
Other	.43 (.21, .87)	.44 (.21, .89)	.35 (.19, .66)	.34 (.18, .65)
**Education**				
Secondary or less	Ref	Ref	Ref	Ref
Vocational / some college	1.03 (.72, 1.47)	1.05 (.73, 1.51)	1.70 (1.19, 2.44)	1.75 (1.21, 2.53)
Bachelor’s degree	1.57 (1.07, 2.30)	1.48 (.99, 2.18)	1.60 (1.11, 2.29)	1.62 (1.12, 2.33)
Graduate degree	1.66 (1.16, 2.36)	1.54 (1.06, 2.23)	1.97 (1.37, 2.83)	2.04 (1.41, 2.95)
**Diagnostic Category**				
Mild Cognitive Impairment	Ref	Ref		
Dementia	.90 (.69, 1.18)	.97 (.74, 1.28)		
**β-Amyloid Scan Results**				
Not elevated	Ref	Ref		
Elevated	.85 (.65, 1.10)	.78 (.60, 1.04)		

### Qualitative sample description

Of the 58 persons with cognitive impairment and 121 care partners who were eligible for the follow-up qualitative study, 39 persons with cognitive impairment and 63 care partners completed the interview, resulting in response rates of 67.2 and 52.0%, respectively; the remainder refused to participate or could not be reached. Of the persons with cognitive impairment who participated in the interviews, 65.8% were men, 86.8% were married or lived with a partner, and 65.8% self-identified as non-Hispanic White. The majority (76.3%) were between 65 and 74 years of age. They were highly educated, similar to the overall sample, with 63.2% having completed graduate education. Slightly more than half (55.3%) of the participants had elevated amyloid levels and the majority (92.1%) had been diagnosed with mild cognitive impairment rather than dementia, which is to be expected given the minimum M-TICS score of 21 (see Table 3) inclusion criterion.

**Table 3:** Descriptive characteristics of participants in follow-up in-depth qualitative interviews, *N* = 100

	Persons with Cognitive Impairment ***N*** = 38	Care Partners ***N*** = 62
**Number of Paired Person with cognitive impairment/Care Partner Dyads in Sample n (%)**^**a**^	30 (78.9)	30 (48.4)
**Age**		
Below 65	N/A	15 (24.2)
65-74	30 (78.9)	32 (51.6)
75+	N/R	15 (24.2)
**Gender**		
Men	25 (65.8)	15 (24.2)
Women	13 (34.2)	47 (75.8)
**Marital Status**		
Married / Cohabiting	33 (86.8)	59 (95.2)
**Race / Ethnicity**		
Non-Hispanic, White	25 (65.8)	34 (54.8)
African American, Hispanic or Other	12 (31.6)	28 (45.2)
**Education**		
Bachelor’s degree or less	13 (34.2)	32 (51.6)
Graduate degree	24 (63.2)	27 (43.5)
**Person with cognitive impairment’s β-Amyloid Results**		
Elevated	21 (55.3)	34 (54.8)
Not elevated	17 (44.7)	28 (45.2)
**Person with cognitive impairment’s Diagnostic Category**		
Mild Cognitive Impairment	35 (92.1)	49 (79.0)
Dementia	N/A	13 (21.0)
**Person with cognitive impairment’s Advance Directive**		
Reported	29 (76.3)	49 (79.0)

*N/A* not applicable, *N/R* not reported because of small cell numbers

^a^Percentages may not add to 100% because of missing values

Half (51.6%) of the care partners were between 65 and 74. Three-quarters (75.8%) were women, 95.2% were married or lived with the person with cognitive impairment, and 43.5% had a graduate education. Due to our purposeful sampling strategy, 45.2% of the care partners who participated in the in-depth interviews identified as Hispanic, Black or African American, or another race other than non-Hispanic White. Approximately half (54.8%) of the care partners cared for a person with elevated amyloid levels. Nearly 80% of the care partners cared for a person with mild cognitive impairment at the time of the scan. All interview participants were recruited from the parent IDEAS study and had already received an amyloid PET scan as research participants [31]. Of the 100 in-depth interview participants, there were 30 dyads in which both members participated.

### Qualitative results

The most salient findings from the qualitative thematic analysis process concerned *the perceived urgency to have or update advance directives, the evolving nature of healthcare preferences, and the overall context of completing an advance directive*. We provide descriptions of each overarching theme below and discuss how they differed along stratifying dimensions including amyloid PET scan results, and race and ethnicity (see Table 4 for additional excerpts).

**Table 4:** Supporting Excerpts from Thematic Analysis

Description	Elevated Amyloid	Not Elevated Amyloid
Theme 1: Perceived urgency to have or update advance directives		
• Certainty / uncertainty about the future • Role of comorbid conditions or other life-threatening situations in making plans • Previous experience with dementia / end-of-life	“It’s in the back of our minds [writing advance directives], not enough to go forward. I think as each year passes, we’re more concerned with that. But we have talked about it a little bit, yes.” (Care partner, other race/ethnicity) “Well, we know what we’re dealing with. We made sure we already had wills and things, but we made sure to update them. We got our affairs in order. We went through everything together, all of our finances, everything together. I remember shortly after the diagnosis, we spent a couple of weeks together in the basement going through everything so that we both be aware of where everything was. We just got everything in order.” (Care partner, non-Hispanic White) “I did one of those [advance directive] when I went in for surgery one time [...] I don’t know why I did it then, but we did it, and I went into surgery. It was for hip replacement” (Person with cognitive impairment, non-Hispanic White) “I lost my brother recently, and he told me I wouldn’t have any decisions to make, everything’s taken care of. Well, he passed away and nobody knew what his things were, his wishes or desires. I was next of kin, so I kind of got adopted into it. Plus we were in different states, so it was not easy. It just reminded us that we better take care of some of this stuff ahead of time.” (Care partner, Hispanic White)	“At the time we did it, it was... We did it I guess about 10 or 12 years ago. And we’re both in [a] reasonable state of mind. It was a process that we felt was necessary, so there was very little anxiety connected with it.” (Person with cognitive impairment, non-Hispanic White) “We haven’t really [discussed end-of-life care preferences]. My husband and I are close, and sometimes he helps me remember, so I rely on him a lot. [...] No. We bought a plot, or a niche, already. We’ve done that, but that’s all. We haven’t even done a will yet”. (Person with cognitive impairment, other race/ethnicity) “It has not been needed. I mean, we have clearly done estate planning and have involved our friends and made sure that we have power of attorneys and we’ve made provisions if (Husband) were to outlive me, of who would manage our finances and any legal things that are needed. […] There’s not a need. He’s doing fine.” (Care partner, non-Hispanic White) “Since COVID began, we started getting serious about our will, and what’s to be done with our remains and etcetera, things about our mortgage. And we’re going to try to leave that type of instruction behind because we know they’re going to be blown over if they have to take over if we both go at the same time.” (Care partner, Hispanic White)
Theme 2: Evolving healthcare preferences		
• Expectations about coping with progressing illness • Empowerment to set boundaries about care • Acceptance of disease process	“We’ve had a living will for a long time, which basically says that if I can’t care for myself, if I can’t care for myself in any way, if I can’t maintain my life in a life full of joy and full of fulfillment, then I don’t want to live. Don’t feed me. I don’t want to be fed. I don’t want to be maintained. […] It would be against my living will. It would be against any will that I have.” (Person with cognitive impairment, other race/ethnicity) “It means that you need to plan now while you still can, and eventually if things degrade your only insurance is already set in motion. Those things you can control. Other things you can’t control and you just accept as part of life.” (Care partner, other race/ethnicity) “It was difficult [discussing care preferences], but we know that eventually we all have to die anyway so we had to do it sometime. It wasn’t too difficult.” (Care partner, Hispanic White)	“So I really haven’t planned on being physically inept to take care of myself. I know one day that may come or maybe I’ll be lucky and like somebody said, they had cancer, they woke up one morning and thought some lucky [guy] had a heart attack last night.” (Person with cognitive impairment, Hispanic White) “The only thing that comes to mind is that we would want to have a cut-off date, or point. We would want to decide when we would change our opinion. I don’t expect him to have Alzheimer’s or to have mental problems that get worse. I simply do not expect it, but if they did, then we would have to discuss it then.” (Care partner, non-Hispanic White) “My mom and I are both what I would consider realists. We realize our days are numbered and we’re both not really afraid of dying from the standpoint that, it’s going to happen.” (Care partner, non-Hispanic White)
Theme 3: Circumstances of writing an advance directive		
• Existing skills and knowledge to plan for future • Costs • Resources such as lawyers, accountant, books and websites • State frameworks (e.g. POLSTs) • Availability of trusted person to designate as surrogate decision-maker	“Well, the process is, we had it done, and now I’m waiting to get some money from my 401 k so I can pay for the attorneys to do this. It’s so expensive. That’s where I’m at right now.” (Person with cognitive impairment, Hispanic White) “We have a POLST, P-O-L-S-T. It’s designed to be on a bright print sheet, so that if anyone comes in the house to try and save you. It says exactly what you want. It’s a little more involved in just the regular one that they have at the hospitals, have you sign or whatever.” (Person with cognitive impairment, Non-Hispanic White) “We haven’t [discussed end-of-life preferences]. Only because this is strong family support and I don’t think I would be thrown to the wolves or left unattended. That is the least of my worry and my concern. So I’m not worried about that. I even have really good friends who I think would step in and step up to help me out if I needed help.” (Person with cognitive impairment, Black/African American)	“I’ve had a will since the beginning, being in the military. Then we got married. We had to put this together and then because of the kind of work we did, having kids we had to have all kinds of stuff put together.” (Person with cognitive impairment, Black / African American) “Well, we’ve done that separately already. Power of attorney and healthcare proxy and so forth. Our accountant actually was the one who prompted us into that”. (Person with cognitive impairment, Hispanic White) “There are educational experiences with our church about this, as well as in Lifelong Learning, which is education through the university for elder people. And so it’s something we talk about easily and share things with other people.” (CARE PARTNER, non-Hispanic White) “No, we don’t have to yet [plan for future]. We will. We will, with our family’s physician when we discuss this.” (Care partner, Black/African American)

There were marked differences between *participants’ perceived urgency to make one’s wishes known through advance directives*. Some participants mentioned the scan results as a trigger to complete or update advance directives: “After [the scan]. Yeah, I started the ball rolling when I realized this wasn’t going to be getting better” (non-Hispanic White person with cognitive impairment, elevated amyloid). However, the level of urgency to document preferences did not align clearly with the scan results, with many participants reporting advance directives written decades before experiencing memory issues: “It was just what you do when you get old” (non-Hispanic White care partner, elevated amyloid); “One thing we have done, is not only because of the scan, but because of general awareness, that we are taking care of our will, advance directives” (care partner of other race/ethnicity, not elevated amyloid). On the other hand, as demonstrated in Table 4, some care partners to persons with elevated amyloid were not yet concerned enough to complete an advance directive: “It’s in the back of our minds, not enough to go forward. I think as each year passes, we’re more concerned with that. But we have talked about it a little bit, yes” (care partner of other race/ethnicity, elevated amyloid). As illustrated in Table 4, the scan results provided some certainty about how severe memory issues could become, prompting them to update documentation: “Well, we know what we’re dealing with. We made sure, we already had wills and things, but we made sure to update them” (non-Hispanic White care partner, elevated amyloid). Even in light of elevated amyloid, other comorbid conditions and participants’ overall age and health were mentioned as drivers of the need to plan for the future: “At the same time, they diagnosed me with Alzheimer’s, I was diagnosed with breast cancer. I just feel like whichever takes me first, I’ll go. I would not like to drag on for years” (person with cognitive impairment, non-Hispanic White). The level of certainty about the progression of memory issues, comorbid conditions, and previous experience caring for a dying person, all shaped participants’ perceived urgency to plan for the future. Many reported having seen first-hand the challenges that come with a person dying without their loved ones having a clear sense of their preferences and expressed a desire to avoid inflicting this on their families:

“It's all done, crossed Ts and dotted Is. I mean, she has the will, estate, trust, funeral arrangements, everything's made. I have copies of everything and deeds are signed to transfer property. [...] I've seen the other side of it where there was no planning and it was just a nightmare” (non-Hispanic White care partner, not elevated amyloid).

The *nature of participants’ healthcare preferences appeared to be affected by the scan results*, whereby those with elevated amyloid envisioned extensive cognitive impairment and dependency when expressing care preferences: “What he would want is, he wants to die before his brain dies. It’s how he puts it. He hopes to die of something else [for] which isn’t easy to plan for” (non-Hispanic White care partner, elevated amyloid). Descriptions of the future were less focused on deteriorating cognition among persons whose test results did not confirm elevated amyloid, as shown in the excerpt in Table 4: “I don’t expect him to have Alzheimer’s or to have mental problems that get worse. I simply do not expect it, but if they did, then we would have to discuss it then” (non-Hispanic White care partner, not elevated amyloid). Advance directives also empowered participants to set boundaries around their future care, something that was very important to them: “I’m still functioning well, or good enough anyway. But yeah, I know where that boundary is, and if my pacemaker ran out of... I’ve got about seven years of battery left on the current pacemaker, and unless I’m doing really well, I’m not going to get the battery replaced” (person with cognitive impairment with elevated amyloid, Hispanic White). These boundaries also included clarifying the care they did want to receive: “Other cancers or some illness that is treatable and I still have reasonably good cognition, I would pursue that. I’m not going to give up saying, ‘Well, I got an amyloid scan. Therefore I want to give up any kind of medical treatments’. No, no, I want to live as long as I can” (non-Hispanic White person with cognitive impairment, elevated amyloid). Several participants also reported acceptance of the potential progression of Alzheimer’s and other disease processes: “I’ve had a good life. I have nothing to complain about. And I’m going to be [over 85 years old] next month. I think I lasted longer than I was supposed to. No complaints. You can’t wish that you’re going to live forever. Not going to happen” (non-Hispanic White, person with cognitive impairment, elevated amyloid).

When describing *the context of completing advance directives*, participants mentioned pre-existing skills and knowledge, the cost of hiring professionals to document plans, the availability of resources such as books, websites, or accountants, the state frameworks guiding these documents, and the availability of trusted persons to designate as surrogate decision-maker. These circumstances were not specific to planning for a future with memory issues, and barriers such as excessive costs were reported by members of underrepresented races and ethnicities, as shown in Table 4: “I’m waiting to get some money from my 401k so I can pay for the attorneys to do this. It’s so expensive” (person with cognitive impairment, Hispanic White, elevated amyloid). Several participants had been healthcare professionals for decades and were well aware of the importance and intricacies of advance care planning: “I’ve been a nurse for [a long time], and I’m very familiar with final wishes” (Black / African American care partner, not elevated amyloid). A number of participants reported not yet having engaged in formal advance care planning and expressed a desire for their medical provider to be involved in this process: “No, we don’t have to yet [plan for future]. We will. We will, with our family’s physician when we discuss this” (Black / African American care partner, elevated amyloid).

## Discussion

### Main findings

As amyloid PET scans become an increasingly common diagnostic tool for Alzheimer’s disease, we sought to better understand the influence of scan results on the likelihood of having an advance directive among Medicare beneficiaries with memory issues of unknown etiology. We found that over 80% of participating dyads reported that the person with cognitive impairment had an advance directive at the time of the CARE-IDEAS telephone survey interview, with over three-quarters reporting concordant answers within dyads. We found no significant association between the likelihood of having an advance directive and either diagnostic category or scan results. This lack of association may be because we only assessed the presence of an advance directive; participants who already had one may still have been more likely to update or discuss it with healthcare providers and next of kin. Our sample also consisted of symptomatic older adults who likely had other neurodegenerative conditions even in the absence of elevated amyloid, which would likely prompt advance care planning. Additionally, participants in qualitative interviews considered wills, trusts, funeral arrangements and long-term care insurance under the umbrella of planning for the future, confirming the ambiguity of this terminology. Concordance between self-reported completion of advance directives and their presence in health records has also been found to be low in prior research [32]. There has been considerable debate about the best process and outcome measures to capture the complexity of planning for the future among older adults with life-limiting illnesses [33].

Having an advance directive in our sample was significantly associated with sociodemographic characteristics (75-84 age, male persons with cognitive impairment and female care partners, non-Hispanic White race/ethnicity, and higher education level), and was more common than the approximately 30% completion rate found in the general US population [34]. This difference is likely attributable to a high level of education among our participants, and to some extent their health status, although adults with chronic conditions do not appear to have much higher rates of advance directives nationally (38.2% compared to the general population rate of 32.7%) [34]. Minority race and ethnicity have been recurrently associated with lower rates of engaging in advance care planning [18, 26]. White race and high education levels have been associated with higher likelihood of advance care planning documentation in previous work among persons with different levels of cognitive impairment [18, 27].

The qualitative results of this study provide additional insight into how participants make sense of amyloid PET scan results and the perceived relevance of this information in planning for the future and documenting their healthcare preferences. Our findings reveal differing levels of perceived urgency to plan for the future, which did not align clearly with amyloid PET scan results, thereby confirming our quantitative findings. Participants discussed this urgency in light of their overall health and comorbid conditions, their previous experiences caring for someone at the end of life, and their certainty or uncertainty about the future. Amyloid scan results seemed more relevant to participants when explaining their evolving healthcare preferences and how they expected to cope with progressing illness. Advance directives also empowered them to set boundaries about the care they did and did not want. These findings resonate with previous research that identified finding the “right time” as a major challenge to engaging in advance care planning among persons with dementia [14, 16–19]. The nature of amyloid PET scan results disclosure discussions may have varied widely across participating sites’ routine practices and has not been studied extensively to date [35], highlighting the uncertainty as to how advance care planning discussions can be or are integrated into clinical practice. Grappling with the looming threat of diminishing capacity is a distinguishing feature of advance care planning for persons with dementia that was also pervasive in our data [13]. Participants discussed a range of resources and potential barriers to engaging in the process of documenting healthcare preferences, most of which were not unique to dementia care and can contribute to explanations of sociodemographic disparities in completion of advance directives in our sample and the larger population.

Although much research and funding has focused on increasing rates of advance directive completion, the overall evidence supporting the effectiveness of advance directives themselves remains mixed. A review of 80 systematic reviews comprising 1660 studies offered limited and low-quality evidence that advance directives and advance care planning positively affect outcomes [36]. Despite the uncertainty as to the value of advance directives and advance care planning overall, systematic reviews demonstrate that among persons with dementia specifically, advance care planning is associated with improved end-of-life outcomes, from decreased hospital admissions to improved concordance and decreased emotional distress for persons with dementia [11, 37]. However, critics of advance care planning as a field and advance directives as a tool argue that there is growing evidence of limited impact on clinical, financial, and patient satisfaction outcomes [33, 38]. These criticisms target advance care planning in older adults generally, and persons with cognitive impairment and their care partners may have unique needs and experiences. Alzheimer’s disease is a progressive, terminal illness, and advance directives support persons with cognitive impairment when they are no longer able to make decisions for themselves, in addition to potentially reducing decisional conflict and supporting care partners as they become surrogate decision-makers [13, 39].

### What this study adds: implications for healthcare practice and directions for future research

Given that considerable uncertainty remains about both the progression of Alzheimer’s disease even in the presence of elevated amyloid, and the efficacy of pharmacological interventions targeting this biomarker [40], determining the role of amyloid PET scan in preparing scan recipients and their care partner for the future is arguably a very important aspect of its potential clinical value. Our results suggest that although elevated amyloid prompted participants to consider progressive cognitive impairment as part of their evolving healthcare preferences, there was still substantial variability in the perceived urgency of documenting these preferences. As we strive to provide increasingly goal-concordant care for persons with dementia, there is growing recognition that healthcare preferences toward the end of life are not static and are influenced by a myriad of factors, and that there are limitations to what advance directives can achieve to support persons with cognitive impairment and their care partners during a complex and emotionally laden time [33]. While an amyloid PET scan may provide persons with cognitive impairment and their care partners with valuable information about the risk of progressive memory impairment, additional research is needed to determine how the general shift to using biomarkers to diagnose Alzheimer’s disease will affect advance care planning and end-of-life care outcomes more broadly.

### Strengths and limitations

This study has some weaknesses. Based on previously documented rates of advance directive completion in the general population with and without chronic conditions [34], we estimated that with an enrollment ratio of 2.2 persons with elevated amyloid for each person with not elevated amyloid in the CARE-IDEAS sample, a larger sample size of 2774 participants would have been necessary for 80% power to detect a statistically significant difference of 5.5% (at alpha = 0.05). The CARE-IDEAS sample was also less diverse and more highly educated than the US population, potentially explaining very high levels of advance directives in our findings. However, we oversampled racial/ethnic minority participants for the qualitative follow-up work in an attempt to capture a broader range of experiences and to hear the perspectives of groups particularly at risk of cognitive impairment. Advance directives were assessed with a single item that did not allow us to examine the timing of their completion, and using a more standard measure such as the ACP Engagement Scale would have improved the generalizability of the quantitative findings [41]. However, in-depth narratives about planning for the future suggest that there are many other meaningful activities in the process of advance care planning that may have been influenced by the scans, such as revisiting plans as one’s health and social network change, updating written documents, and discussing preferences with trusted persons and potential surrogates, and the qualitative nature of the interviews allowed participants to describe their engagement with the aspects that they found meaningful. We have limited information as to the exact content of clinical discussions between patients and their providers discussing the amyloid PET scan results and how these specific discussions may have prompted advance care planning. We also completed the quantitative survey work before the coronavirus pandemic, while in-depth interviews were conducted by telephone at times during serious infection waves, which may have shaped participants’ perceptions of the need to plan for the future and to document their healthcare preferences. The study nonetheless has notable strengths, including a mixed-methods research design that gives a voice to healthcare users. The focus on symptomatic participants with memory issues of unknown etiology also increases the clinical relevance of our findings given that they represent a likely target population for amyloid screening in clinical practice, beyond enrollment in disease-specific trials [7].

## Conclusions

In this sequential, mixed-methods research study, persons with cognitive impairment with elevated amyloid were not more likely than those without elevated amyloid to report an advance directive, although the qualitative data suggest that expectations of progressive memory issues still shaped their healthcare preferences. There remained substantial variability in the perceived urgency of documenting these preferences on the basis of participants’ overall health and their previous experiences with end-of-life care. There were also notable racial and ethnic disparities in having advance directives and in the resources available to support this documentation process.

## Supplementary Information

**Additional file 1.**

## Data Availability

The datasets used and analyzed during the current study are available in the Brown Digital Repository (10.26300/87rp-a575) which includes a CARE-IDEAS codebook, and a PDF file with a description of the software used and syntax used for data cleaning and the final analytical models.
