# Supplementary material for: Advance directives among cognitively impaired persons who had an amyloid PET scan and their care partners: a mixed-methods study
Source: BMC Palliat Care. 2022 Nov 6;21:194. doi: 10.1186/s12904-022-01082-4 (PMC9638311; doi:10.1186/s12904-022-01082-4)
Supplement: Supplementary file 1 — Additional file 1. [file 12904_2022_1082_MOESM1_ESM.pdf]

Appendix

Patient Version - Interview Guide for CARE IDEAS Qualitative Telephone Conversation (Note that sub-questions are potential probes)

1) Please tell me how you decided to have an amyloid PET scan.
Who recommended the scan?
(If a doctor recommended): How long had you been seeing this doctor?
What prompted the first visit to the doctor?
What did you hope to learn from the scan?
What were the main reasons you decided to have the scan?
Could you tell me more about any doubts or concerns you had about having the scan?
(At any time) Thank you, that’s very helpful.

2) How were the amyloid PET scan results communicated to you?
Did you receive a phone call or did you have an in-person visit? Did you talk with a physician or other health professionals?
What information was given to you when discussing the results?
What do you think about the way this information was given to you? (enough information, enough time, etc.) I’d like to know a bit more about that.

3) How do you understand the results of the amyloid PET scan?
What does it mean to have a diagnosis of Alzheimer’s [use participants’ own words for probes]?
In your opinion, what does this mean for your future?
What questions do you have about the result?

4) How did you feel about the results?
How has this changed over time?

5) How have the scan results affected your plans for the future?
What else do you need to know to plan for your future?
What about involving family members in decisions and in planning for your care?
What about writing a living will or discussing end-of-life care preferences?
(If person says they already have a living will) What was that process like?

6) If your health were to get worse, please describe your preferences for care.
If you developed another illness, to what extent would you pursue diagnostic tests and treatments? Where would you want to be cared for? Have you made arrangements for this (e.g. LTC insurance)?
Have you talked about your preferences with your care partner?
What else comes to mind?

7) How is the health care that you are receiving consistent with your care preferences?
Tell me about care that you’d like to change.
You said you had a preference for XYZ, how is your care matching this?
I know these questions sound alike. I am trying to understand what it's been like for you in detail.

8) What would you tell a friend/family member about whether they should have an amyloid PET scan? Would you recommend it?

Please tell me more. We're interested in your reasons why or why not, and what you think the value of this test is.

9) What else would you like to tell us about your experience having an amyloid PET scan and receiving the test results?

Care Partner Version - Interview Guide for CARE IDEAS Qualitative Telephone Conversation
(Note that sub-questions are potential probes)

1) Please tell me how you and the person you are caring for decided to have an amyloid PET scan.
Who recommended it?
(If a doctor recommended): How long had he/she been seeing this doctor?
What prompted the first visit to the doctor?
What did you hope to learn?
What were the main reasons he/she decided to have the scan?
Could you tell me more about any doubts or concerns you or he/she had about having the scan?

2) How were the amyloid PET scan results communicated to you and the person you are caring for?
Did you receive a phone call or did you have an in-person visit? Did you talk with a physician or other health professionals?
What information was given when discussing the results?
What do you think about the way this information was given to you? (enough information, enough time, etc.)

3) How do you understand the results of the PET scan?
What does it mean to have plaque / a confirmed diagnosis of Alzheimer's [use participants' own words for prompts]?
In your opinion, what does this mean for his/her future?
What questions do you have about the result?
How different is your understanding of the scan from the person you are caring for?

4) How do you feel about the results?
Has this changed over time? I'd like to know a little bit more about that.

5) How have the scan results affected his/her plans for the future?
What else do you need to know to plan for the future?
What about involving family members in decisions and in planning for care needs?
What about writing a living will or discussing end-of-life care preferences? What was that process like?

6) If [the patient's] health were to get worse, what do you think are his/her preferences for care?
If he/she developed another illness, to what extent would he/she pursue diagnostic tests and treatments? Where would he/she want to be cared for? Have you made arrangements for this (e.g. LTC insurance)?

Have you talked about these preferences together?
What else comes to mind?

7) How is the health care that the person you are caring for is receiving consistent with his/her care preferences? Tell me about care that you'd like to change.
You mentioned that there was a preference for XYZ, how is the care matching that?

8) What would you tell a friend/family member about whether they should have an amyloid PET scan? Would you recommend it?
Please tell me more. We're interested in your reasons why or why not, and what you think the value of this test is.

9) What else would you like to tell us about your experience as the care partner of a patient who received an amyloid PET scan and the test results?
